# Evolving nutrition therapy in cystic fibrosis: Adapting to the CFTR modulator era

**DOI:** 10.1002/ncp.11332

**Published:** 2025-06-18

**Authors:** Kay Vavrina, Tara B. Griffin, Angel M. Jones, Terri Schindler, Trang N. Bui, Senthilkumar Sankararaman

**Affiliations:** ^1^ University Health San Antonio Texas USA; ^2^ University of Minnesota Medical Center Minneapolis Minnesota USA; ^3^ Children's Hospital Los Angeles Los Angeles California USA; ^4^ University Hospitals Cleveland Medical Center Cleveland Ohio USA; ^5^ UT Health San Antonio San Antonio Texas USA; ^6^ Cleveland Clinic Children's Hospital Cleveland Ohio USA

**Keywords:** aging, cardiometabolic risk, CF‐related diabetes, CF‐related liver disease, CFTR gene mutations, CFTR modulators, cystic fibrosis (CF), elexacaftor/tezacaftor/ivacaftor (ETI), exocrine pancreatic insufficiency, fat‐soluble vitamin, gastrointestinal manifestations, ivacaftor, legacy CF diet, nutritional therapy, obesity, overweight, vanzacaftor

## Abstract

Cystic fibrosis transmembrane regulator (CFTR)–directed therapies, such as modulators, have transformed the medical management of people with CF, resulting in better lung function, weight, and body mass index in recent years. With improved nutrition status in people on CFTR modulators, the emphasis on a high‐energy, high‐fat diet (the legacy CF diet) is declining, with an increased focus on a healthy diet. The increased survival and median predicted age of people with CF have created a need for more attention to metabolic diseases, including hypertension, dyslipidemia, and cardiovascular diseases. The effects of modulators on extrapulmonary manifestations associated with CF, such as CF‐related diabetes, CF hepatobiliary involvement, gastrointestinal tract disorders, and pancreatic manifestations, are currently unknown. Approximately 95% of people with CF qualify for treatment with a CFTR modulator. This review discusses the basics of CFTR gene mutations and changes in nutrition status related to treatment with CFTR modulators.

## INTRODUCTION

Mutations in the cystic fibrosis transmembrane regulator (CFTR) gene result in the absence or decreased activity of the CFTR protein, an epithelial transporter of chloride and bicarbonate.^1^ CF is an autosomal recessive disease affecting approximately 90,000 people worldwide.^2^ CF is a multisystem disease affecting the respiratory system, pancreas, gastrointestinal (GI) tract, biliary tract, reproductive system, and electrolyte homeostasis.^1^, ^3^ Malfunctions in or a lack of the CFTR protein result in the production of viscous, sticky mucus in the lungs and other organ systems, leading to increased pulmonary infections, impaired nutrient absorption, GI symptoms, sinus disease, and male infertility.^4^ Historically, improvement in respiratory management and nutrition therapy (pancreatic enzyme replacement therapy and supplemental nutrition) has been the cornerstone for improved outcomes and reduced morbidity in CF.^5^

In recent years, CFTR‐directed therapies such as genetic modulator therapy have transformed the medical management of people with CF (pwCF), resulting in improved pulmonary function, weight, and body mass index (BMI).^6^, ^7^, ^8^, ^9^ However, the overall effects of modulators on extrapulmonary manifestations associated with CF, such as the GI tract, CF‐related diabetes (CFRD), and CF hepatobiliary involvement (CFHBI), are currently unknown.^9^, ^10^ This review aims to discuss the basics of CFTR gene mutations and the effect of CFTR modulators on nutrition status and various extrapulmonary manifestations of CF.

### Basics of nutrition challenges and complications in pwCF

Malnutrition and CF have been correlated since the disease was first recognized.^5^, ^11^ Exocrine pancreatic insufficiency (EPI), which results in impaired secretion of bicarbonate and digestive enzymes, increased energy expenditure, altered fatty acid metabolism, CFRD, altered bile acid physiology, small intestinal bacterial overgrowth, and increased inflammation may each contribute to the multifactorial root cause of CF‐related malnutrition.^5^ Pancreatic insufficiency, which is found in approximately 80% of pwCF, contributes to the malabsorption of macronutrients and fat‐soluble vitamins. A correlation has been found in multiple studies between increased resting energy expenditure (REE) and decreased pulmonary function, which has led clinicians to support recommendations well above the recommended daily energy allowance for pwCF.^11^ It is well established in the literature that optimal nutrition status is associated with improved pulmonary function.^12^ To meet elevated REE requirements, a high‐energy, high‐fat diet has typically been recommended to all pwCF and is often referred to as “the legacy CF diet.” The dietary focus has not been on diet quality but rather on the high energy intake to maintain weight status.^13^, ^14^ This legacy high‐energy, high‐fat diet has been a cornerstone of CF nutrition care since the landmark study by Corey et al., which outlined dietary differences, weight, and pulmonary outcomes in pwCF managed in Toronto and Boston.^15^ Energy intake recommendations are 110%–200% of the standard population‐required needs and lack restrictions on saturated fat, sugar, or salt.^16^ Limited evidence is available identifying the specific dietary requirements of pwCF and current guidelines are based on expert consensus.^16^, ^17^, ^18^, ^19^, ^20^

The CF Foundation (CFF) and an international multidisciplinary workgroup consisting of representatives of the European Society for Clinical Nutrition and Metabolism (ESPEN); European Society of Pediatric Gastroenterology, Hepatology and Nutrition (ESPGHAN); and European CF Society (ECFS) each have published guidance including BMI targets for pwCF.^14^, ^18^, ^21^ BMI goals are ≥ 22 kg/m^2^ and ≥ 23 kg/m^2^ for adult women and men, respectively. In pediatric pwCF, a weight‐for‐length at the 50th percentile for infants and children aged ≤2 years and a BMI at the ≥50th percentile for children aged 2 to 18 years are categorized as adequate nutrition status.^14^, ^21^

To better understand the nutrition patterns of adults with CF, researchers conducted a comprehensive systematic review in 2023. The authors knew of no other literature reviews focusing on dietary intake and composition. They found suboptimal diet quality compared with diets in healthy Americans, with higher intakes of fat and a lack of fiber‐containing foods such as whole grains, fruits, and vegetables.^16^ Another study used the Healthy Eating Index to measure diet quality in pwCF.^13^ Diet quality was lower in healthy pwCF than in the general population. PwCF specifically have lower intakes of fruits, vegetables, greens, and legumes, resulting in lower intakes of fiber.^13^, ^16^ The historical focus has been weight gain or maintenance rather than chronic disease prevention, with more recent studies documenting dyslipidemia, low high‐density lipoprotein cholesterol levels, and elevated triglycerides in some pwCF.^13^, ^22^, ^23^

### CFTR biochemistry

**Figure 1:**
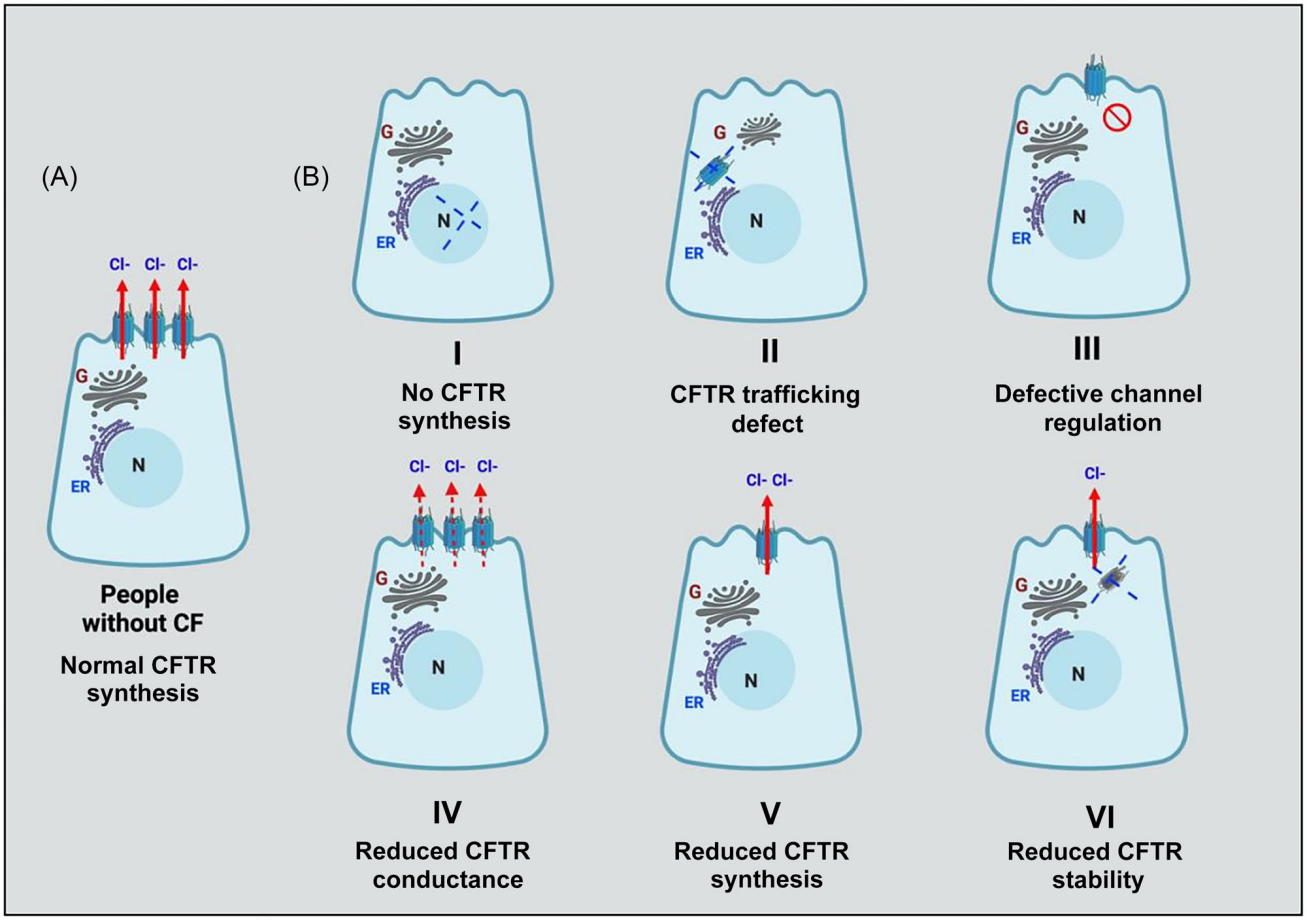
Illustration of CFTR function. (A) People without CF. (B) CFTR class mutations in people with CF. CF, cystic fibrosis; CFTR, cystic fibrosis transmembrane regulator. Created in BioRender. Used with permission under BioRender's Academic License. © Sankararaman, S. 2025. https://biorender.com/j00t453.

More than 2100 genetic variants in the CFTR gene have been identified, with approximately 700 variants reported to be disease‐causing.^24^ Six classes have been determined to define the various disease‐causing mutations (Figure 1). Class I mutations, affecting approximately 22% of pwCF, are also known as nonsense mutations or deletions. Class I mutations are associated with severe disease resulting in little to no CFTR protein production owing to the interruption of transcription.^1^, ^3^, ^4^, ^24^ Deletion of phenylalanine at the 508 position of the CFTR protein (F508del), a class II or trafficking defect, is the most common CF‐causing mutation, resulting in a protein that is misfolded and degraded at the cell surface.^1^, ^24^, ^25^ Approximately 85% of pwCF in the US have at least one copy of F508del. This mutation has been the focus of drug development over the last several decades.^3^ Gating (class III) mutations result in a malfunction at the channel gate of the cell, despite adequate production and trafficking of the CFTR protein.^1^, ^4^ The conductivity of the channel gate is altered in class IV mutations, affecting chloride permeability.^4^ Class III and IV mutations are less common, each affecting 6% of pwCF. Class V and VI defects produce CFTR protein with decreased amounts at the surface of the cell and decreased stability, respectively.^1^

### Modulators targeting CFTR

CFTR modulators are orally administered medications that improve CFTR function, improving protein folding, trafficking, and function. These medications bind to the CFTR protein either during or after protein processing, ultimately increasing the amount of CFTR protein available at the cell surface. Studies have reported improvement in lung function and an improvement in GI complications associated with CF after initiation of CFTR modulator therapy.^4^ CFTR modulators are categorized into potentiators, correctors, amplifiers, and stabilizers.^4^, ^11^, ^24^ Correctors and potentiators are currently used in pwCF who have eligible mutations.^4^

Potentiators, such as ivacaftor (IVA), improve the channel opening and are thus helpful for pwCF who have gating mutations (class III mutations) such as G551D.^4^ Correctors, such as lumacaftor (LUM) and tezacaftor (TEZ), improve the trafficking, folding, and expression of the CFTR protein. The increased stability of the CFTR protein in pwCF who are eligible for correctors improves CF symptoms.^4^, ^24^ Combination therapy, which involves combining a corrector with a potentiator, has been shown to increase the amount of CFTR protein at the surface of the cell for people with homozygous F508del mutations.^24^ The approval of triple combination therapy, elexacaftor/TEZ/IVA (ETI), increased access to CFTR modulator therapy for pwCF carrying at least one copy of F508del. Overall, ETI has been shown to improve lung function, BMI, and quality of life.^11^, ^25^ Even though CFTR modulator therapy is associated with decreased rates of pulmonary exacerbations, the long‐term effect and rate of progression of disease pathology have not yet been determined.^25^ More recently, a triple combination medication, vanzacaftor‐TEZ‐deutivacaftor (VTD), has been marketed for pwCF who have at least one copy of F508del.^26^ VTD differs from previous iterations of modulator therapy in that only once‐daily dosing is required compared with twice daily. The results of two multicenter safety and efficacy studies showed that VTD improved pulmonary function similar to ETI through week 24 of treatment. This novel CFTR modulator treatment shows promise for improving respiratory function while reducing pill burden for pwCF; however, its long‐term impact on nutrition status and extrapulmonary comorbidities is unknown Table 1 details the different types of currently available modulators with their mechanism of action, eligible age of the patients, and the potential side effects of the modulators.^27^

**Table 1:** CFTR modulators, which class of CFTR defects they correct, and the ages, dosages, and potential side effects.

CFTR modulator and the mechanism of action	Year of first approval	Class of CFTR defects	Eligible age	Commonly noted side effects	Serious side effects that require baseline evaluation and regular monitoring during therapy
Ivacaftor (potentiator)	2012	Currently, people with 87 mutations are eligible. Most mutations belong to class III/IV	>1 month	Headache, upper respiratory tract infection symptoms, stomach ache, diarrhea, and rash	‐ Drug‐induced liver injury (manufacturers recommend evaluation of liver function. Liver enzymes, such as ALT, AST, alkaline phosphatase, and bilirubin, need to be evaluated before initiation of modulators and monitored serially thereafter)^a^ ‐ Hypersensitivity reactions including allergies ‐ Cataract
Lumacaftor (corrector)/ivacaftor (potentiator)	2015	Two copies of F508del mutation	>1 year		
Tezacaftor (corrector)/ivacaftor (potentiator)	2018	Two copies of F508del mutation	>6 years		
Elexacaftor (corrector)/tezacaftor (corrector)/ivacaftor (potentiator)	2019	At least one copy of F508del mutation	>2 years		
Vanzacaftor (corrector)/tezacaftor (corrector)/deutivacaftor (potentiator)	2024	At least one copy of F508del mutation	>2 years		

*Note*: For more information on the medications listed, please see https://www.vrtx.com/en-us/medicines/our-approved-medicines, https://www.kalydeco.com, https://www.orkambi.com, https://www.symdeko.com, https://www.trikafta.com, and https://www.alyftrek.com.

Abbreviations: ALT, alanine aminotransferase; AST, aspartate aminotransferase; CFTR, cystic fibrosis transmembrane regulator.

^a^ Modulator therapy may need to be interrupted if the enzymes raise significantly (ALT or AST >5 times the upper limit of normal or ALT or AST >3 times the upper limit of normal with bilirubin >2 times the upper limit of normal) or clinical manifestations indicative of liver failure, such as jaundice, right upper quadrant pain, nausea, vomiting, altered mental status, and ascites. Modulators should not be used in patients with severe liver impairment (Child‐Pugh Class C) and are not recommended in patients with moderate liver damage (Child‐Pugh Class B) unless benefit clearly outweigh the risk.

### The changing face of nutrition therapy in CF with CFTR modulators

Advances in general CF care and the impact of CFTR modulators have drastically decreased the incidence of malnutrition in pwCF, whereas rates of overweight and obesity have increased from 12.8% in 1999 to 31.4% in 2019.^7^ Improvements in weight and BMI were reported as primary outcomes in approval studies for IVA (approved by the US Food and Drug Administration [FDA] in 2012).^28^ Approvals for subsequent medications (LUM/IVA in 2015, TEZ/IVA in 2018, and ETI in 2019) focused on less obvious nutrition improvements, such as better appetite and pancreatic wellness.^29^ The expanded approval of ETI for pwCF with one F508del mutation associated with one gating or residual function mutation and eventually to those with one copy of F508del led to the availability of CFTR modulator therapy for >90% of pwCF.^25^

IVA treatment, the first modulator available to pwCF, was shown to increase BMI‐for‐age *z* scores in children with CF and the G551D mutation after treatment for 48 weeks compared with placebo. Twenty‐four weeks of LUM/IVA treatment led to a 0.2‐kg/m^2^ increase in BMI in children aged ≤12 years with two copies of F508del when compared with placebo, whereas treatment with TEZ/IVA for 24 weeks did not result in a significant increase in BMI when compared with the placebo group.^7^

ETI treatment has resulted in a more profound effect on weight gain in pwCF. In adults with CF, BMI has been shown to increase substantially.^8^, ^30^ The PROMISE^31^ study, a 56‐center prospective observational study, reported an increase in mean BMI from 23.1 ± 4.0 kg/m^2^ at baseline to 24.5 ± 4.6 kg/m^2^ after 6 months of ETI treatment. In children and adolescents with CF aged 2 to 19 years between 2005 and 2019, median BMI percentiles increased from the 46th percentile to the 61st percentile, and adults aged 20 to 40 years improved, with median BMI results from 21.2 to 23.1 kg/m^2^.^20^ Increases in overweight and obesity in pwCF were observed in 2020 (26.7% and 10.3%, respectively) relative to 14.3% of adults with CF who were overweight or obese in 2000.^20^ In general, pwCF who have exocrine pancreatic sufficiency tend to have a higher BMI than those with EPI.^30^ Normal weight obesity, characterized by a normal weight and BMI with an increased body fat percentage, is correlated negatively with lung function and has been reported in pwCF by Alvarez et al.^30^, ^32^, ^33^ ETI has had the most significant impact on BMI and weight‐for‐length percentiles.^34^ As the CF population grows older, cases of increased cholesterol and coronary artery disease have been reported in this group, although more prospective research is needed to identify contributing factors.^30^, ^35^, ^36^ Considering the increases in overweight and obese pwCF, there is an increased concern for metabolic and age‐related conditions associated with excess intake of saturated fat, trans fat, added sugar, and other dietary components traditionally prevalent in the CF diet.^30^

### Nutrition challenges after modulator therapy/compared with “the legacy CF diet”

**Figure 2:**
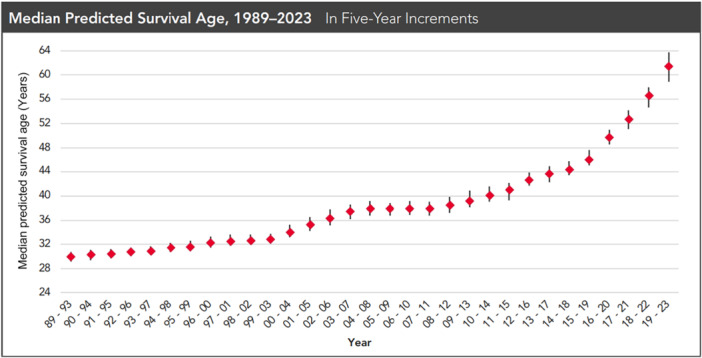
Data from the Cystic Fibrosis Foundation Patient Registry, 2023 Annual Data Report. Used with permission from the Cystic Fibrosis Foundation.

PwCF are living longer than ever, creating nutrition challenges. Data collected from the CFF patient registry report predicts the median survival age of patients born in 2023 is >60 years (Figure 2).^37^ In comparison, the 2014 annual report indicated a median survival age of 39.3 years.^37^ Improvement in survival is primarily because of working closely with registered dietitians to optimize nutrition status and the development of CFTR modulator therapy.^6^, ^17^, ^38^

In general, aging populations often experience a decline in health, financial burdens owing to the cost of medical care, and a decrease in quality of life.^39^, ^40^ Greater life expectancy correlates with an increased risk of developing age‐related chronic diseases, including cardiovascular and cerebrovascular diseases, along with certain types of cancers and disability.^40^, ^41^ Diet‐related conditions, such as hypertension, dyslipidemia, obesity, diabetes, and insulin resistance, contribute to chronic disease development and may be preventable through diet modifications.^39^, ^40^, ^42^ Trends in the 2023 CFF patient registry report revealed a steep rise of 41.4% of the adult CF population with a BMI in the overweight and obese categories, according to the US Centers for Disease Control and Prevention.^37^ In a recent survey involving 75 CF providers, 29% of adult providers prescribed lipid‐lowering medications, and 54% started antihypertensives in the past year.^23^

There are limited studies on pwCF and the development of age‐related chronic diseases, such as cardiovascular and cerebrovascular diseases or cancer, owing to the limited life expectancy before CFTR‐directed modulator treatment.^36^ The 2020 Academy of Nutrition and Dietetics (AND) evidence‐based nutrition guidelines for pwCF recognized increasing obesity rates and a lack of recommendations for maintaining a healthy weight owing to limited clinical evidence. More specifically, no clinical or observational studies were identified that studied nutrition intake or macronutrient distribution in pediatric or adult patients.^20^ ESPEN/ESPGHAN/ECFS guidelines published in 2024 reported decreased malnutrition risk with a concurrent steady increase in obesity rates over the past two decades.^18^ This review found suggestive evidence of a relationship between overweight and obesity and health risks similar to those of the general population. Updated recommendations to prevent obesity and comorbidities in pwCF, such as hypertension, hypercholesterolemia, and diabetes, were published.^18^ Additionally, recent guidance from the CFF suggests that weight, BMI, and energy goals be individualized, taking medications, including CFTR modulator therapy, nutrition status, disease severity, and physical activity, into consideration.^20^

### CFTR modulator therapy and fat‐soluble vitamins

There is some evidence that CFTR modulator therapy has resulted in increased serum levels of fat‐soluble vitamins, particularly vitamin A; however, increases in vitamins E and D have also been reported.^31^, ^43^, ^44^, ^45^, ^46^, ^47^ Hypervitaminosis A has been associated with papilledema after the initiation of ETI in some cases.^48^ Vitamin E toxicity is not commonly observed but may be associated with excessive bleeding and intracranial hemorrhage.^49^, ^50^ Serum vitamin D levels may fluctuate after initiation. Fabricius et al.^44^ reported an initial increase in mean serum vitamin D levels from 27.26 ± 9.05 μg/L 4 weeks before beginning CFTR modulator therapy to 29.33 ± 9.29 μg/L 4 weeks after beginning therapy. This was followed by a decrease to 25.36 ± 8.70 μg/L at 3 months after the initiation of CFTR modulator therapy, followed by an increase to 28.6 ± 11.85 μg/L at 12 months after start.^44^ Similar results were reported by Carnovale et al.^43^ Serum vitamin D (ng/ml) levels decreased to 25.25 (16.33–33.03) at 3 months after modulator initiation from baseline levels of 29.40 (22–34.80).^43^ At 12 months, serum vitamin D levels increased to 27.6 (22.9–34.17).^43^ Modulator‐specific multivitamin formulations with decreased quantities of vitamins A and E have been marketed; however, there is no guidance supporting the use of these novel formulations or making adjustments to traditional dosing recommendations.^51^ Large, longitudinal studies are needed to understand the mechanisms behind these fluctuations and to support guidance for how and when to support vitamin supplementation in this era. Additionally, increased monitoring of serum fat‐soluble vitamin levels is warranted, considering recent reports of toxicities associated with increased levels.^31^, ^43^, ^44^, ^45^, ^46^, ^47^

### CFRD

CFRD is associated with increased morbidity and mortality and contributes to lung function decline, weight loss, and worsened mental health and is a predominant complication of CF.^52^, ^53^, ^54^ CFRD is a disease complication unique to pwCF. Although it does share some features with type I and type II diabetes, its pathophysiology differs in both functional and structural aspects.^55^ Within the pancreas, functional impairment of beta cells is likely due to the presence of CFTR gene expression in the ductal cells.^55^, ^56^ Islet cells are influenced by ductal cells via paracrine signaling, possibly affecting insulin release.^55^ A defective CFTR protein may also cause structural problems within pancreatic islet cells, resulting in the decreased mass of these cells over time. People with advanced CF disease have about 50% fewer insulin‐producing beta cells even if they do not have a diagnosis of CFRD.^37^ Other factors contributing to CFRD may include inflammation, hereditary features, and nutrition status.^37^, ^55^, ^56^

The CFF patient registry annual report for 2023 indicates that 29.3% of adults (≥18 years) and 4.2% of children and adolescents (<18 years) have been diagnosed with CFRD.^37^ This is also described as 18.9% of all reported complications, making CFRD the most common nonpulmonary comorbidity associated with CF.^37^, ^57^ CFRD rarely occurs in infancy, but the prevalence increases with age. The dominant feature of CFRD is insulin insufficiency; decreased insulin secretion is even seen in pwCF who do not have a diagnosis of CFRD.^52^, ^57^ Insulin sensitivity is usually normal; however, insulin resistance may be present owing to acute illness, stress, or aging or in the setting of obesity.^57^, ^58^ Decreased insulin secretion has been observed in two phases: reduced early‐phase insulin secretion (<30 min after eating/glucose ingestion) and a blunted and/or delayed second‐phase insulin release.^59^ Similar to type I and II diabetes, CFRD is associated with microvascular complications including retinopathy, nephropathy, neuropathy, and gastroparesis.^57^, ^60^ CFRD can have additional health implications related to the disease process of CF, including a decline in pulmonary function, an increase in pulmonary exacerbations, compromised nutrition status, and an increased risk of depression.^53^

Routine screening for CFRD is important, as typical symptoms of diabetes may be mild or absent in this population. Current clinical practice guidelines recommend a 2‐h oral glucose tolerance test performed annually starting at age 10 years.^52^, ^57^, ^61^ A 2‐h plasma glucose of ≥200 mg/dl can be used to make a diagnosis of CFRD. Hemoglobin A1c (HbA1c) levels may be falsely decreased in pwCF, and fasting hyperglycemia tends to be a late finding.^59^ However, the standard criteria of HbA1c ≥6.5 or fasting hyperglycemia >125 mg/dl are valid for a diagnosis of CFRD, when observed.^52^ Specialized screening recommendations exist for pwCF in a state of acute illness, during pregnancy, before transplantation, and for those receiving continuous enteral nutrition support.^52^, ^57^, ^61^ Currently, the use of a continuous glucose monitor (CGM) is not a validated method for the screening and diagnosis of CFRD.^62^ However, CGM data are beneficial in identifying early changes in glycemia that may serve as an indication for expedited CFRD testing.^62^, ^63^

Glycemic goals for CFRD do not differ from those established for type I and type II diabetes. Current guidelines from the American Diabetes Association advise fasting/preprandial plasma glucose levels of 70–126 mg/dl, 2‐h postprandial levels of 90–180 mg/dl, and maintaining HbA1c ≤7.^64^ Self‐monitoring of glucose levels should be performed multiple times per day with a minimum frequency of three time points or as prescribed to the individual by their care team.^52^ Both fingerstick blood glucose monitors and CGMs are valid for self‐monitoring in the CFRD population.^52^, ^62^

Insulin replacement is the primary treatment for CFRD and has been shown to improve pulmonary function and nutrition and support overall better glycemic control.^52^, ^57^ Insulin may be given as a combination of both short‐ and long‐acting forms in a basal‐bolus system or be prescribed subjectively to best fit the individual's glucose management needs. Current dosing recommendations for CFRD state that most pwCF require 0.5–0.8 units of insulin/kg body weight/day when in their usual state of health.^57^ In periods of illness, stress, or growth, insulin requirements may increase acutely and should be adjusted. In general, insulin should be prescribed in the largest amount the patient can safely tolerate without hypoglycemia to counteract the catabolic effect of insulin insufficiency. However, high doses of insulin may increase the risk of hypoglycemia, which is a common complication of CFRD.^56^ Patients with recurrent hypoglycemia may benefit from the use of CGM technology, either with or without a paired insulin pump.^62^

Historically, dietary recommendations for CFRD have not varied greatly from a typical diet prescription for pwCF.^20^, ^57^ Current guidelines recommend that carbohydrates should total 45%–50% of the total daily energy intake; however, it is advised that this proportion of macronutrient division be individualized based on treatment goals.^52^ Adjustment in carbohydrate intake may be appropriate, particularly when using carbohydrate counting or as a strategy for the management of reactive hypoglycemia. Dietary fat intake has traditionally not been limited for people with CFRD because of low rates of cardiovascular disease.^65^ However, modern treatments for pwCF are associated with increasing rates of overweight/obesity, which may have future implications on the dietary management of CFRD.^66^ Many advances in the care of pwCF have been made since the publication of CFRD guidelines by the CFF, which suggests that additional research on CFRD and other CF‐related comorbidities in the era of CFTR modulator therapy is warranted.^52^

New technologies and treatments for CF and CFRD have the potential to improve health outcomes and lessen the burden of CFRD management. The International Society for Pediatric and Adolescent Diabetes (ISPAD) guidelines support the use of CGM in individuals with CFRD using insulin for blood glucose management.^57^ Despite the ability of CGM to identify blood glucose abnormalities associated with Β cell dysfunction in cross‐sectional and retrospective studies, large multicenter longitudinal studies are needed before CGM is recognized as a screening/diagnostic tool for CFRD. ISPAD guidelines support the use of insulin pumps, with and without the concurrent use of CGM.^57^ Modern devices, including automated insulin delivery systems and the iLet Bionic Pancreas (Beta Bionics), are being evaluated for use in CFRD.^67^, ^68^

Incretin‐based therapies, which are commonly used in the type II diabetes population, may also have applications in the treatment of CFRD. Although limited data are currently available, glucagon‐like peptide‐1 receptor agonists (GLP‐1 RAs) have been observed to support glycemic control in people with CFRD while reducing their prescribed insulin requirements.^69^, ^70^ Additional benefits of GLP‐1 RAs may include improvement in lung function and promotion of weight loss for individuals with obesity. However, this class of medications can cause side effects that may be undesirable in CF, including delayed gastric emptying, severe constipation, pancreatitis, and undesired weight loss.^71^

Finally, CFTR modulator therapy has the potential to change outcomes for people with CFRD. Early studies indicate some improvement in glycemia for patients taking the ETI medication.^72^, ^73^ However, the impact of CFTR modulator therapy on CFRD is not yet completely understood, and more studies are needed to provide future guidance.

### Exocrine pancreatic and GI involvement in CF

PROMISE‐GI^9^ analyzed a subset of data reporting the effect of ETI on GI symptoms. The 56‐center prospective, observational study used validated questionnaires and laboratory tests to assess GI signs and symptoms before and 6 months after ETI therapy initiation.^9^ The overall impact of ETI treatment on GI symptoms was small, with little effect on bloating, rectal symptoms, intestinal inflammation, and pancreatic insufficiency.^9^ Improvement in BMI was reported in the PROMISE^9^ study after treatment with ETI for 6 months. There is also emerging evidence of positive effects of ETI on appetite, and oral intake reduces energy expenditure and the coefficient of fat absorption.^66^

**Figure 3:**
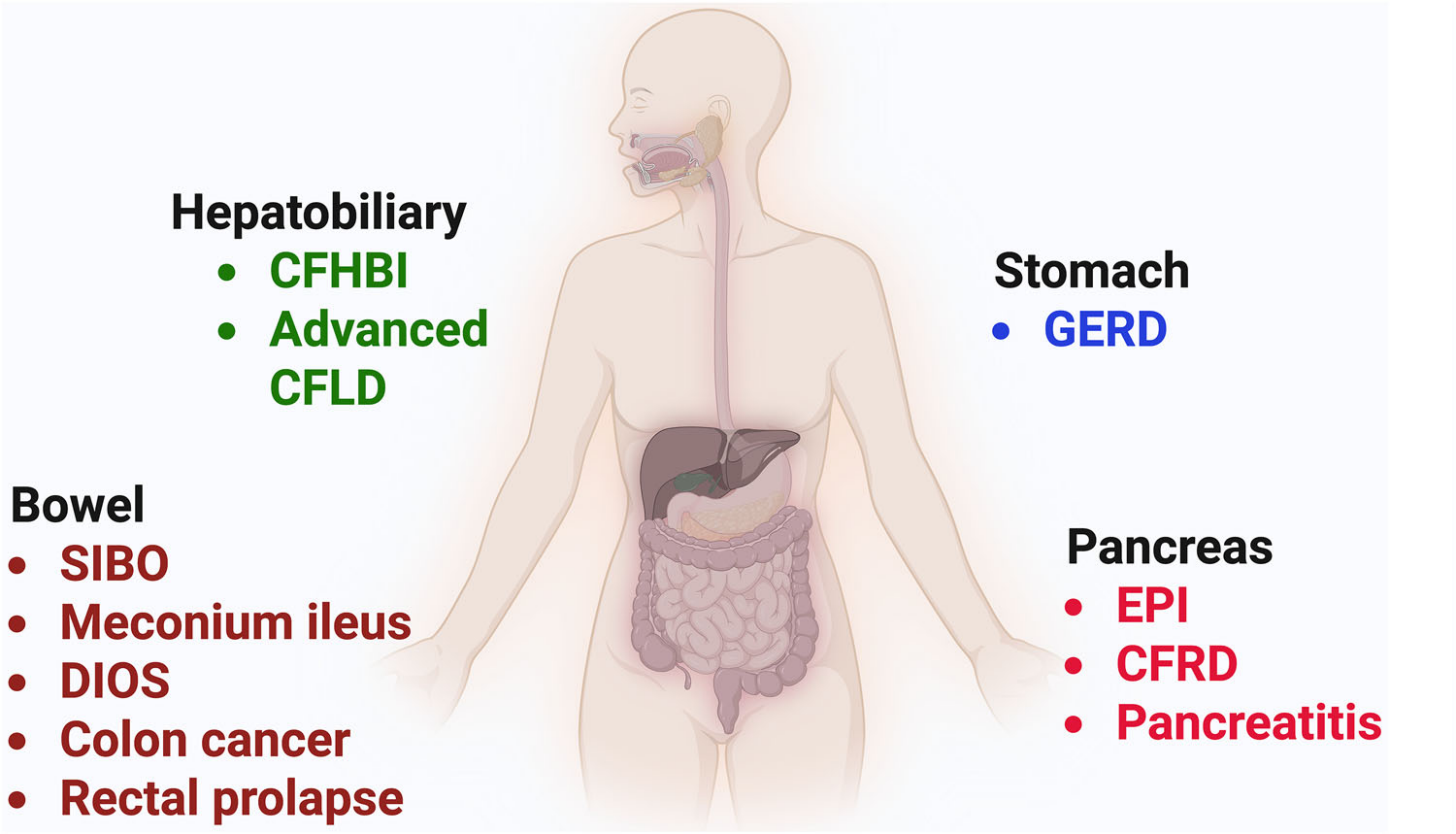
Cystic fibrosis gut manifestations. CFHBI, cystic fibrosis hepatobiliary involvement; CFLD, cystic fibrosis–related liver disease; CFRD, cystic fibrosis–related diabetes; DIOS, distal intestinal obstruction syndrome; EPI, exocrine pancreatic insufficiency; GERD, gastroesophageal reflux disease; SIBO, small intestinal bacterial overgrowth. Created in BioRender. Used with permission under BioRender's Academic License. © Sankararaman, S. 2025. https://biorender.com/8rotgw5.

Once thought irreversible and permanent, improvement in pancreatic function was noted in many patients after IVA treatment.^67^, ^68^, ^69^ However, the PROMISE‐GI study did not show significant improvement of pancreatic function (measured by fecal elastase‐1) in children with CF aged >12 years post‐ETI treatment.^9^ With the use of ETI in the younger population, more patients may demonstrate improvement in pancreatic function.^70^ Interestingly, patients with improvement in pancreatic function are at risk of developing acute or chronic pancreatitis and should be evaluated and managed accordingly (Figure 3).^70^

### CF with hepatobiliary involvement

In CF, hepatobiliary involvement can range from cholelithiasis, hepatic steatosis, and focal liver fibrosis to more advanced CF‐related liver disease (aCFLD), which encompasses multilobular cirrhosis and its complications. These may include portal hypertension and decompensated liver failure requiring liver transplantation. In 2024, a position paper from ESPGHAN and the North American Society of Pediatric Gastroenterology, Hepatology and Nutrition (NASPGHAN) and another publication from the CF liver disease guidelines committee both emphasized the standardized approach to the nomenclature of hepatobiliary involvement in CF.^74^, ^75^ “The presence of nodular liver, advanced fibrosis (F4), multilobular cirrhosis with/without portal hypertension, or noncirrhotic portal venopathy are classified as aCFLD.”^74^ The rest of the spectrum of involvement was categorized as CFHBI, which refers to having “one (or more) of the following without any features of aCFLD such as hepatomegaly, liver fibrosis (<F4), increased liver stiffness by elastography (<F4), hepatic steatosis, focal biliary cirrhosis, cholestasis, persistent (>3–6 months) elevated serum liver function tests (any level above the upper limit of normal), abnormal liver imaging, cholelithiasis, sclerosing cholangitis, or hepatolithiasis.”^74^ Single‐center studies evaluated the changes in liver biomarkers following treatment with ETI.^76^, ^77^, ^78^ Currently, there is a lack of evidence to substantiate that the use of CFTR modulators improves or worsens the outcome of CFHBI or aCFLD.^74^, ^79^ Given the potential possibility of hepatotoxicity with the use of CFTR modulators, caution should be exercised when people with aCFLD take these medications. Current management of aCFLD is based on symptomatic management, including nutrition support and management of complications such as portal hypertension and liver transplantation in people who have liver decompensation. Figure 3 lists the gastrointestinal and nutritional manifestations of CF.^75^

### Nutrition in the current era of CFTR modulators

As the landscape of CF treatment is evolving with the approval for the use of CFTR modulators in younger people, we can expect more data on safety and efficacy. Near‐normal life expectancy in a population with a historically decreased life span can be achieved, particularly in those pwCF treated with CFTR modulators early in life. However, there are still many questions on nutrition intervention. Also, there remain about 10% of pwCF who carry CFTR variants that are not suitable targets for CFTR modulators. Additionally, some patients develop adverse effects from CFTR modulators and cannot continue this therapy. An individualized approach addressing all the nutrition needs is needed to optimize outcomes in pwCF. Drug development continues to address all underlying mechanisms and symptoms of CF with the goal of improved treatments for CF disease pathologies.^25^

### Nutrition guidance moving forward

The 2024 ESPEN/ESPGHAN/ECFS guidelines on CF nutrition care of infants, children, and adults acknowledged a change in the CF nutrition landscape along with ample evidence that dietary patterns in the general population that maintain a healthy weight can prevent chronic disease, includes consuming fruits and vegetables with whole grains and limiting added sugar, salt, and saturated fat.^18^, ^39^ Updated CF recommendations changed from the high‐energy, high‐fat diet to a diet similar to that recommended to the general population. The traditional legacy diet recommendations lack evidence for increased energy intake, as well as high‐fat/high‐salt diets for many pwCF on highly effective modulators. An individualized nutrition therapy approach is needed based on underlying nutrition status, modulator therapy status, pancreatic status, personal risk factors, family history, and the presence of multiple comorbid conditions such as dyslipidemia, diabetes, hypertension, and aCFLD or lung disease.

Nutrition counseling is essential to address the challenges associated with CFTR therapies in the CF population.^19^, ^22^, ^38^ PwCF have been encouraged to consume the “CF legacy diet” with a focus on a high‐energy, high‐fat, high‐salt diet, creating an ingrained behavior in many. Behavioral change can be complex and challenging for providers and patients.^22^, ^80^, ^81^ A 2023 study examined CF clinicians’ current views on managing CFTR modulator–related weight gain and increased BMI issues. Clinicians felt CF‐specific patient data were needed before recommending changes to current recommendations.^81^ Clinicians describe feeling ill prepared to address the issue without data showing the potential consequences of overweight and obesity as a risk for chronic disease or potential impact on pulmonary function.^38^, ^80^ This reluctance may be, in part, due to the strong historical correlation between weight and lung function.^6^, ^14^, ^15^ Given the lack of clinical evidence to direct CF‐specific recommendations for these new challenges, expertise in behavior modification and nutrition guidelines is essential.^38^, ^80^ Medical nutrition therapy should be individualized, incorporating the needs and goals of patients while considering the current therapeutic burden on pwCF and their families.^18^, ^20^, ^38^ Support is needed from the entire multidisciplinary team to deliver consistent messaging and guidance.^22^, ^38^

## CONCLUSION

The medical management of CF has evolved with the advent of CFTR modulator therapies.^6^, ^7^, ^8^, ^9^ Dramatic improvements in lung function, with increased rates of overweight and obesity, have caused researchers to speculate on how changes in the traditional high‐fat/high‐energy diet may impact age‐related comorbidities not previously seen in this population.^35^ The known major comorbidities associated with CF, such as CFRD and aCFLD, will likely remain problematic as pwCF age.^35^, ^82^ Nutrition management of CF is a work in progress. Clinicians will need to pivot to address diabetes with a pathophysiology that more closely resembles that used for type 2 diabetes. The management of aCFLD will evolve as clinical studies reveal how the long‐term use of CFTR modulators affects the development of CFHBI and aCFLD. As pwCF taking CFTR modulators age, c‐morbidities associated with advanced age, such as cardiovascular disease, dyslipidemia, and malignancies, are becoming more common.^83^ Further research is needed to better understand the most effective management for CF‐related comorbidities in a post–CFTR modulator world.

## AUTHOR CONTRIBUTIONS

Kay Vavrina contributed to the conception of the manuscript, analysis of the content, and drafting of the manuscript and agrees to be accountable for all aspects of the work ensuring integrity and accuracy. Tara B. Griffin contributed to the conception, design, and drafting of the manuscript and gave final approval. Angel M. Jones contributed to conception, design, and drafting of the manuscript and gave final approval. Terri Schindler contributed to the conception and design of the manuscript, critically revised the manuscript, and gave final approval. Trang N. Bui contributed to the conception and design of the manuscript, critically revised the manuscript, and gave final approval. Senthilkumar Sankararaman contributed to conception of the manuscript, analysis of the content, and drafting of the manuscript and agrees to be accountable for all aspects of the work ensuring integrity and accuracy.

## CONFLICT OF INTEREST STATEMENT

Senthilkumar Sankararaman serves as a consultant for Nestlé Health Science. Kay Vavrina serves on the speaker's bureaus of Alcresta Therapeutics, Abbvie, Nestlé Health Science, and DCI. The remaining authors declare no conflict of interests.
